# Toll-like Receptor-4 Activation Boosts the Immunosuppressive Properties of Tumor Cells-derived Exosomes

**DOI:** 10.1038/s41598-019-44949-y

**Published:** 2019-06-11

**Authors:** Rossana Domenis, Adriana Cifù, Daniele Marinò, Martina Fabris, Kayvan R. Niazi, Patrick Soon-Shiong, Francesco Curcio

**Affiliations:** 1 Dipartimento di Area Medica (DAME), Università degli Studi di Udine, Udine, Italy; 2 Istituto di Patologia Clinica, Azienda Sanitaria Universitaria Integrata di Udine (ASUID), Udine, Italy; 3 NantBioScience, Inc Culver City, CA 90232 USA

**Keywords:** Cancer microenvironment, Immunology

## Abstract

The biology of tumor-derived exosomes (TEX) is only partially understood and much remains to be studied in order to define the effect that the tumor microenvironment or the activation of tumor cells exerts on their composition and functions. Increased expression and activity of toll-like receptor 4 (TLR4) in chronic infectious and inflammatory conditions is related with cancer progression: its activation induces an inflammatory signaling that increases the tumorigenic potential of cancer cells promoting their immune evasion. We investigated the immune modulatory properties of TEX released upon cell TLR4 activation, and we found that, although differences were observed depending on the type of the tumor, the treatment influences TEX composition and boosts their immunosuppressive ability. Our results suggest that the activation of TLR4 supports tumor progression by stimulating the release of more effective immunosuppressive exosomes, which allow tumor cells to escape immune surveillance and probably even play a role in the metastatic process.

## Introduction

Exosomes are population of nanometer-sized extracellular vesicles that originate from the multivescicular endosome and are released by all cells. Exosomes play a critical biological role in cell-cell interaction and through the transfer of functionally-active proteins, lipid and nucleic acid, they are able to influence the behavior of recipient cells, influencing a broad variety of cellular activity in health and disease^1,2^.

Although it is widely recognized that tumor cells actively produce, release and utilize exosomes to promote tumor growth and metastasis^3,4^, the biology of tumor-derived-exosomes (TEX) is not completely understood, and much remains to be study to define the molecular and genetic mechanisms by which exosomes operate in cancer^3^.

The immunological activities of TEX influences immune regulation mechanisms, including modulating antigen presentation, immune activation, immune suppression, immune surveillance, and intercellular communication^5,6^. Their molecular cargo partially derived from the surface of parent tumor cells and is enriched in immunosuppressive proteins (i.e. FasL, PD-L1, inhibitory cytokines, PGE_2_) as well as tumor-associated-antigens and co-stimulatory molecules^7^. Consequently, the molecular profile endows TEX with a dual ability of mediating either immune suppression or immune stimulation, presumably depending on the microenvironment influences on their parental cells^3^. It appears, that in the tumor microenvironment, where tumor cells are actively engaged in suppression of anti-tumor immunity and activities of immune cells are blocked, TEX are primarily utilized to disable anti-tumor immune effector cells and promote tumor escape from immune control^8,9^.

Inflammation and immunosuppression are well known to be involved in cancer progression. Chronic inflammation promotes tumor development, progression, and metastatic dissemination, and above all plays an important role in the suppression of antitumor immunity^10^. As research on extracellular vesicles progresses, it is becoming clear that part of these interactions could be mediated by exosomes.

Evidences from recent studies suggest that enhanced expression^11–14^ and activity of toll-like receptor 4 (TLR4) in chronic infectious and inflammatory conditions are correlated with cancer progression^15,16^. The activation of the TLR4 pathway induces an inflammatory signaling that increase the tumorigenic potential of cancer cells and promotes their immune evasion^17,18^. Therefore, an significant role is attributed to different molecules of host origin that have lately arisen as potential endogenous ligands of TLR4. These proposed endogenous molecules include different components of the extracellular matrix, intracellular proteins, or modified lipids or lipoproteins^15^. Interestingly, many of them are proposed to activate TLR4 without having any substantial structural similarity to endotoxin, its natural ligand^15^.

Many studies have been aimed at identifying the correlation between chronic inflammation mediated by TLR4 and cancer development and progression, but the role of TEX has not yet been investigated. The aim of this study is to explore the immunosuppressive properties of cancer cell-derived exosomes released upon TLR4 activation by its direct ligand lipopolysaccharide (LPS). Our results show that the activation of TLR4 supports tumor progression by promoting the release of more effective immunosuppressive exosomes, although differences were observed depending on the origin of the tumor.

## Methods

### Cell culture and LPS treatment

The human tumor cell lines SW480 (primary colon adenocarcinoma), SW620 (metastatic colon adenocarcinoma), MDA-MB-231 (metastatic breast adenocarcinoma) and U87-MG (glioblastoma) were purchased from Sigma-Aldrich. Cells were grown in two different Dulbecco’s modified essential media (DMEM high glucose for SW480 and SW620 and DMEM low glucose for MDA-MB-231 and U87-MG) (Sigma-Aldrich) supplemented with 10% FBS (Gibco) and 1% penicillin/streptomycin solution (Gibco) at 37 °C with 5% CO_2_ in humidified air.

To activate TLR4 cells were incubated with [1 µg/ml] LPS (*E. coli* 055:B5 LPS, Sigma-Aldrich) for 24 hours, washed three times with PBS and then culture medium was replaced with fresh medium supplemented with 10% certified exosomes-free serum (Gibco). After 24 hours, supernatants were collected and stored at −20 °C until use, while cells were harvested, and their proliferation/viability was determined by the trypan blue exclusion assay.

Cell homogenates were analyzed for TLR4 expression by immunoblotting and the human anti-TLR4 (1:500, Cell Signaling) and anti-β-actin (1:10,000, Sigma-Aldrich) were used as primary antibodies.

All methods of analysis were carried out in accordance with the relevant guidelines and regulation with appropriate quality control.

### Exosomes isolation and characterization

Exosomes were isolated from the supernatants of the cell lines with the polymer precipitation method with ExoQuick-TC (System Biosciences) as previously described^19^. The number of exosomes was determined using the Exocet kit (System Biosciences), according to the manufacturer’s instructions, and size distribution was evaluated by nanoparticles tracking analysis (NTA) with the Nanosight LM10 system (Malvern Instrument Ltd.) equipped with a 532 nm laser.

Cells lysate, exosomes and Exoquick-derived supernatants were analyzed for the expression of exosomal markers and contaminants by immunoblotting, as previously described^19^. Specific primary antibodies against CD9 (1:1000, System Bioscience), CD63 (1:1000, LS Bio), CD81 (1:500, Abcam), TSG101 (1:500, Abcam), calnexin (1:1000, Enzo Life Technologies), GRP94 (1:1000, Genetex) and RISC (1:1000, Abcam) were used. As a secondary antibody we used an anti-IgG antibody conjugated with horseradish peroxidase.

Exosomes (2 × 10^9^) were also purified by immunoaffinity Exo-Flow kit (System Biosciences)^19^, stained with Exo-FITC provided by the kit or specific monoclonal antibodies anti-CD81 FITC (Biolegend), anti-CD63 FITC (Santa Cruz), anti-CD9 PE (eBiosciences), anti-MICA/B Alexa Fluor 488 (Invitrogen), anti-ULBP-1 APC (Invitrogen) and anti-TGFβ1 PE (eBiosciences) and analyzed by flow cytometry.

The expression of TGF-β isoforms was also quantified using the TGF-β Magnetic Luminex Performance Assay kit (R&D Systems). Exosomes (1 × 10^8^) were activated with HCl, neutralized and diluted in RD6-50 buffer, according to the manufacturer’s instructions, and subsequently analyzed on a Bio-Plex 200 system (Bio-Rad).

### Labelling of tumor exosomes

To investigate the ability of CD14+ monocytes and CD3+ T cells to internalize tumor exosomes, vesicles were labelled with DiD (Invitrogen), as previously described^20^. Briefly, 1 × 10^10^ exosomes were resuspended in PBS and stained with 5 μM DiD for 30 minutes at 37 °C. DiD labelled exosomes were incubated with 2 × 10^5^ isolated PBMCs for 6, 14, 24, 18 hours and then cells were analyzed by flow cytometry by gating either on CD14+ or CD3+ PBMCs.

### Isolation of monocytes and T cell population

For the *in vitro* experiments, samples of whole blood from healthy donors were collected in EDTA-tubes by the Department of Transfusion Medicine (University Hospital of Udine). All healthy donors gave their informed consent to this study according to the Declaration of Helsinki and to the Italian legislation (Authorization of the Privacy Guarantor No. 9, 12^th^ of December 2013). Only anonymized leftovers samples from routine clinical practice were used and their use was not subjected to ethics review, according to the International Standard ISO 15189 “Medical Laboratories. Particular requirements for quality and Competence, 2nd Ed. 2007” and Food and Drug Administration OBM Control No 0910-0582 “Guidance of informed consent for *in vitro* diagnostic device studies using leftover human specimen that are not individually identified”.

Human peripheral blood mononuclear cells (PBMCs) were separated by density gradient centrifugation (700 × g for 20 minutes) on a Ficoll Hypaque (Millipore) and resuspended at 1 × 10^6^ cells/ml in RPMI 1640 complete medium supplemented with 10% FBS, 1% glutamine, 1% Na pyruvate, 1% non-essential aminoacid, 1% penicillin/streptomycin, 1% Hepes (all from Sigma-Aldrich). To remove the exosomal fraction present in FBS, serum was always ultracentrifuged for 4 hours at 100,000 × g^19,20^.

CD16+/CD56+ NK, CD4+ T cells and monocytes were purified from PBMCs by negative selection using immunomagnetic beads (StemCell Technologies), according to the manufacturer’s instructions. The efficiency of the purification was over 95% as assessed by staining with specific antibodies and flow cytometric analysis (FACSCalibur)

### Functional studies

#### CFSE proliferation assay

The proliferation of T cells were analyzed by CFSE proliferation analysis, as previously described^20^. To determine the immunomodulatory effect of exosomes on PBMCs, 2 × 10^5^ cells resuspended in 200 μl of medium were preincubated for 24 hours with 5 × 10^9^, 5 × 10^8^, 5 × 10^7^ and 5 × 10^6^ exosomes produced by tumor cells, then stimulated with pre-bound 0.5 μg/ml anti-CD3 (clone OKT3, eBiosciences) and 0.5 μg/ml anti-CD28 (clone CD28.6, eBiosciences). After 3 days, *in vitro* stimulated PBMCs were stained with anti-CD3 APC, anti-CD4 APC or anti-CD8 APC (all from eBiosciences) and cell proliferation was tested by flow cytometry.

#### Treg cell induction

Isolated CD4+ T lymphocytes were incubated with 5 × 10^8^ exosomes and stimulated with anti-CD3 and anti-CD28 in the concentrations described above. The recombinant IL2 at the concentration of 250U/ml was also added as a stimulus. After 3 days, the percentage of FoxP3+ regulatory T cells in the total population of stimulated CD4+ cells, was determined by CD25 surface staining and FoxP3 intracellular staining using Foxp3/Transcription Factor Staining Buffer Set (eBiosciences)^20^. In a separate set of experiments, SW480-, SW620- and U87-MG-derived exosomes were pre-incubated for 15 min at 37 °C with 0.5 µg/ml of anti-TGFβ1 neutralizing antibody (R&D system) before adding to cells.

#### NKG2D expression levels

Resting PBMCs or purified NK cells obtained from healthy donors were co-incubated with or without 5 × 10^8^ exosomes for 24 hours, then cells were stained with anti NKG2D APC and anti-CD8 FITC or anti-CD16 FITC/anti-CD56 PE (all from eBiosciences) and tested by flow cytometry. In a separate set of experiments, SW480- and SW620-derived exosomes were pre-incubated for 15 min at 37 °C with 5 µg/ml of anti-MICA/B neutralizing antibody (R&D system) before adding to cells.

#### Production of Inflammatory proteins

Monocytes (2 × 10^5^) were incubated with 5 × 10^8^ exosomes for 48 hours, then supernatants were collected, centrifuged for 10 minutes at 14,000 × g and concentration of inflammatory proteins (sE-Selectin, GM-CSF, ICAM-1/CD54, IFNγ, IFNγ, IL-1α, IL-1β, IL-4, IL-6, IL-8, IL-10, IL-12p70, IL-13, IL-17A/CTLA-8, IP-10/CXCL10, MCP-1/CCL2, MIP-1α/CCL3, MIP-1β/CCL4, sP-Selectin, TNFα) was assessed by 20-multiplex ELISA kit (ProcartaPlex, ThermoFisher Scientific). To ensure that inflammatory proteins were not previously present in our exosome preparation, their presence was also measured directly in the samples of exosomes.

### Exosomal RNA isolation and RT-PCR

Exosomal RNA cargo was extracted from 1 × 10^10^ exosomes, using mirVana PARIS Kit (Life Technologies), adding cel-miR39 spike-in as exogenous control (100 pM) (ThermoFisher Scientific), as previously described^19^. The extracted RNA was stored at −80 °C until use. Its quantity and quality have been evaluated with the NanoDrop™ 1000 Spectrophotometer (ThermoFisher Scientific).

The relative concentrations of miRNAs (has-miR-21-5p, has-miR-155-5p, and has-miR-34a-5p) were assessed using the TaqMan® Advanced miRNA Assays (ThermoFisher Scientific), according to the manufacturer’s instructions.

Real-time reaction was performed on the Applied Biosystems QuantStudio 3 System. MiRNA relative concentrations were normalized using relative standard curve method obtained by serial dilutions of cel-miR39 (1 nM–100 fM)^19^.

### Statistical analysis

The data are expressed as mean ± standard deviation. Statistical analysis has been performed using the GraphPad Software (version 7). Data were tested for normal distribution using the Kolmogorov-Smirnov test. Numerical data were processed for significance by one-way analysis of variance followed by the Bonferroni or Dunnett post test. A value of P-value of 0.005 was considered significant.

## Results

### Effect of TLR4 activation by LPS on tumor cell

**Figure 1:**
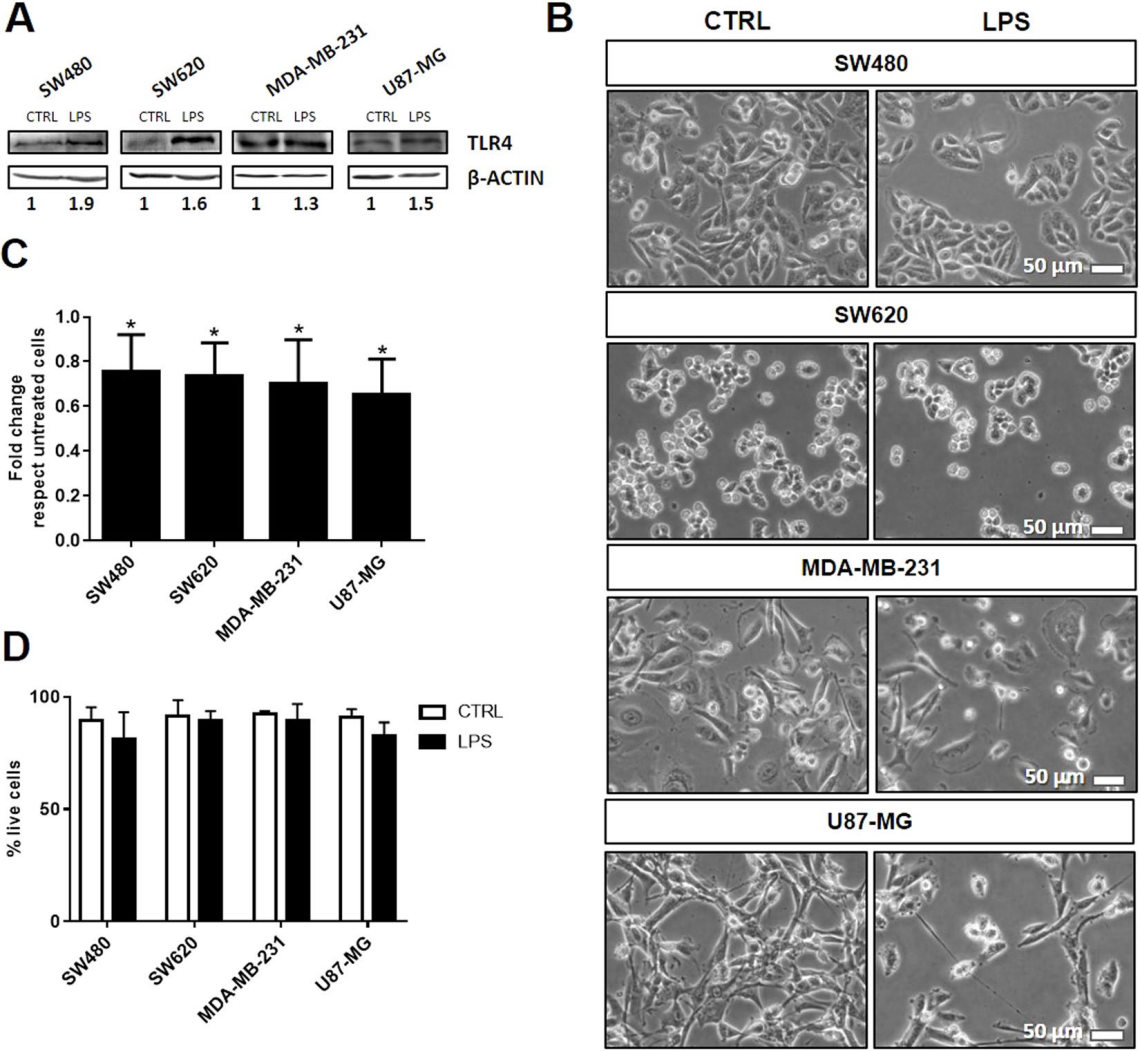
Activation of TLR4 by LPS inhibits proliferation of tumor cells. (**A**) Expression of TLR4 and β-actin in cell homogenates by immunoblotting analysis. (**B**) The figure shows representative phase contrast images (scale bar 50 µm) of SW480, SW620, MDA-MB-231 and U87-MG cells incubated with LPS for 24 hours. (**C,D**) Cell proliferation and viability data are shown. They were determined by trypan blue exclusion assay. Data are shown as mean (n = 15) ± SD. *P < 0.05, compared to untreated cells.

To evaluate whether TLR4 activation may affect morphology, proliferation and viability of tumor cells, cells were cultured for 24 hours in the presence of 1 µg/ml LPS, a TLR4 specific ligand. Results are reported in Fig. 1A through 1D. We show that the treatment with LPS upregulate the expression of TLR4, as verified by immunoblot analysis of cell homogenates (Fig. 1A). We observed that the treatment did not induce morphological changes (Fig. 1B), while the cell proliferation was decreased by about 25% in all the analyzed cell lines (Fig. 1C). Moreover, we observed that cell viability was not affected by treatment with LPS (Fig. 1D).

### Validation of exosomes released by tumor cells upon TLR4 activation by LPS

Tumor cells were pre-treated with LPS, then an enriched fraction of exosomes was isolated from the cells supernatants by polymer precipitation method.

**Figure 2:**
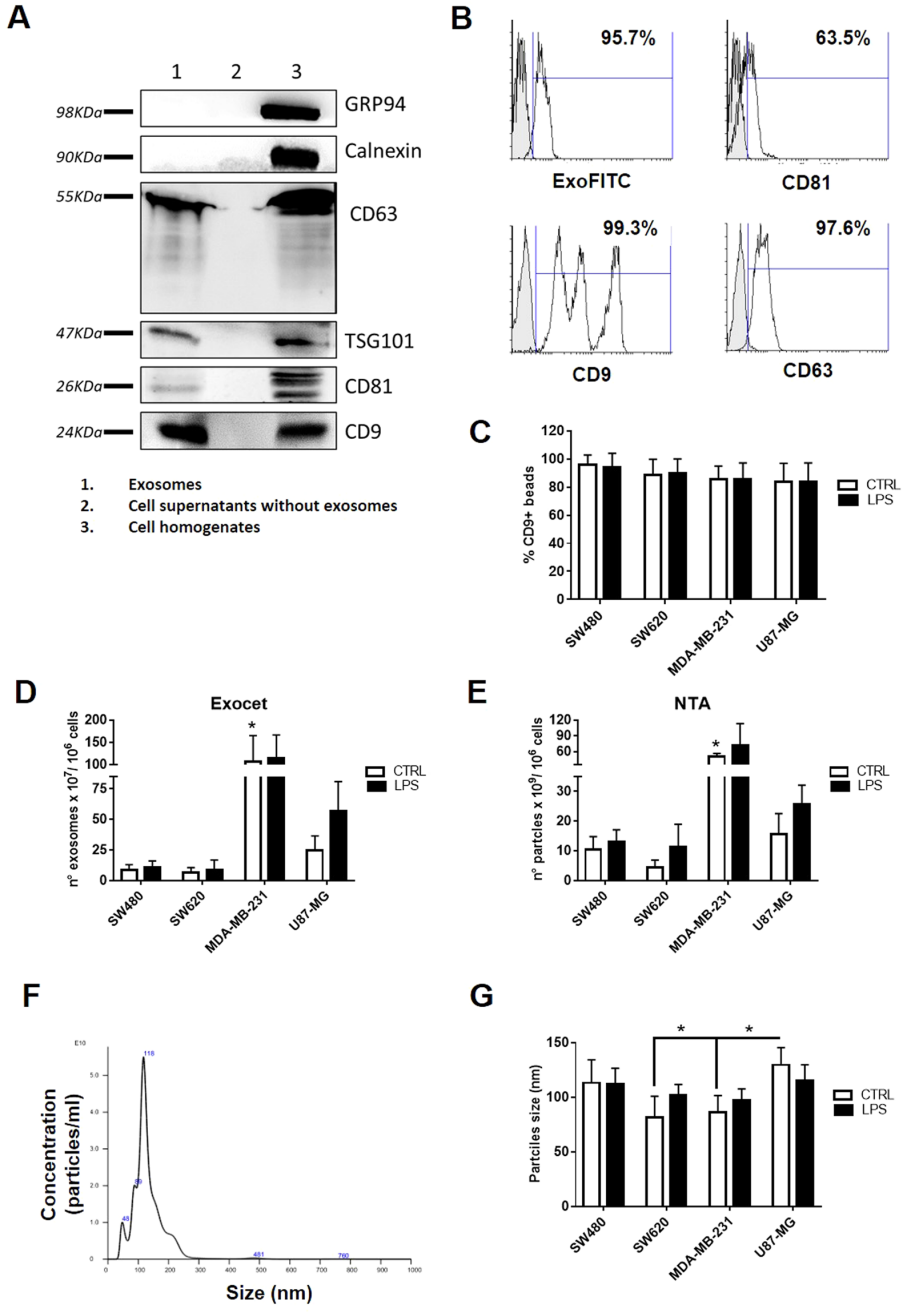
Characterization of exosomes released by tumor cells. (**A**) Immunoblot analysis of SW480-derived exosomes, Exoquick-derived supernatants and cells homogenates probed for the indicated proteins. (**B**) Exosomes were coupled to ExoFlow beads, stained with ExoFITC dye or specific monoclonal antibody for CD81, CD9, and CD63 and analyzed by flow cytometry. The antibodies (white peak) were compared with their appropriate isotype control (grey peak). Histograms from one representative experiment are shown. (**C**) The histogram represents the percentages of CD9-positive beads bound to exosomes released by unstimulated (CTRL, white column) or LPS-activated cells (LPS, black column). Data are shown as mean (n = 4) ± SD. (**D**) The number of exosomes was estimated measuring the enzymatic activity of the exosomal AChE enzyme by Exocet kit (D) or by nanoparticle tracking analysis (NTA) by LM10 Nanosight. (**E**) Data are shown as mean (n = 8) ± SD. *P < 0.05 compared to SW480- SW620 and U87-MG-derived exosomes. (**F,G**) The particles size distribution was evaluated by Nanosight and a representative graph of frequency size distribution of SW480-derived exosomes is shown. Data are shown as mean (n = 8) ± SD. *P < 0.05.

As shown in Fig. 2A and Supplementary Fig. 1A, exosomes and cell lysates expressed the specific exosomal markers CD63, TSG101, CD81 and CD9, while no signal was detected with the Exoquick-derived supernatants, which were used as negative controls. To exclude any possible contaminations in our exosomes preparations, we also evaluated the expression of proteins associated with subcellular compartments. Our data showed lack of calnexin and GRP94 (endoplasmic reticulum proteins), indicating a successful enrichment. The expression of exosomal markers was further confirmed by flow cytometric analysis of exosomes coupled to Exo-Flow beads. As shown in Fig. 2B, beads bound to exosomes expressed high levels of CD81, CD9 and CD63 and reacted with the ExoFITC reagent. It is interesting to note that the expression profile of CD9 highlighted the presence of three distinct exosomes subpopulations and its expression did not change after the treatment with LPS (Fig. 2C).

The concentration of exosomes released by tumor cells was determined measuring the activity of AChE with the Exocet kit (Fig. 2D) and by Nanoparticle tracking analysis (NTA) (Fig. 2E and Supplementary Fig. 1B). These quantification methods provided different absolute values, probably due to the different principle on which the respective analyzes are based. Specifically, NTA analysis reported a concentration of about 100 times higher than that measured with the Exocet kit. However, both quantification methods showed that cellular activation with LPS by stimulation of TLR4 did not influence the number of exosomes released, although a slight non-significant increase, was observed for exosomes produced by U87–MG. It is interesting to report that untreated MDA-MB-231 cells released the highest number of exosomes. The number of exosomes used as treatment for subsequent experiments was quantified by Exocet.

As reported in the dimensional profile of the representative NTA analysis (Fig. 2F), tumor exosomes are detected as nanoparticles with dimensions comparable to those described for physiological exosomes (range from 50 to 120 nm in diameter). Specifically, the average size of the collected vesicles was 113 ± 21.1 nm for SW480-derived exosomes, 81.5 ± 19.7 for SW620-derived exosomes, 86.4 ± 15.1 for MDA-MB-231-derived exosomes and 129 ± 16.1 for U87-MG. Finally, the size of exosomes was not influenced by LPS treatment of producing cells (Fig. 2G).

### Exosomes released upon LPS treatment inhibit T cell proliferation in a dose-dependent manner

**Figure 3:**
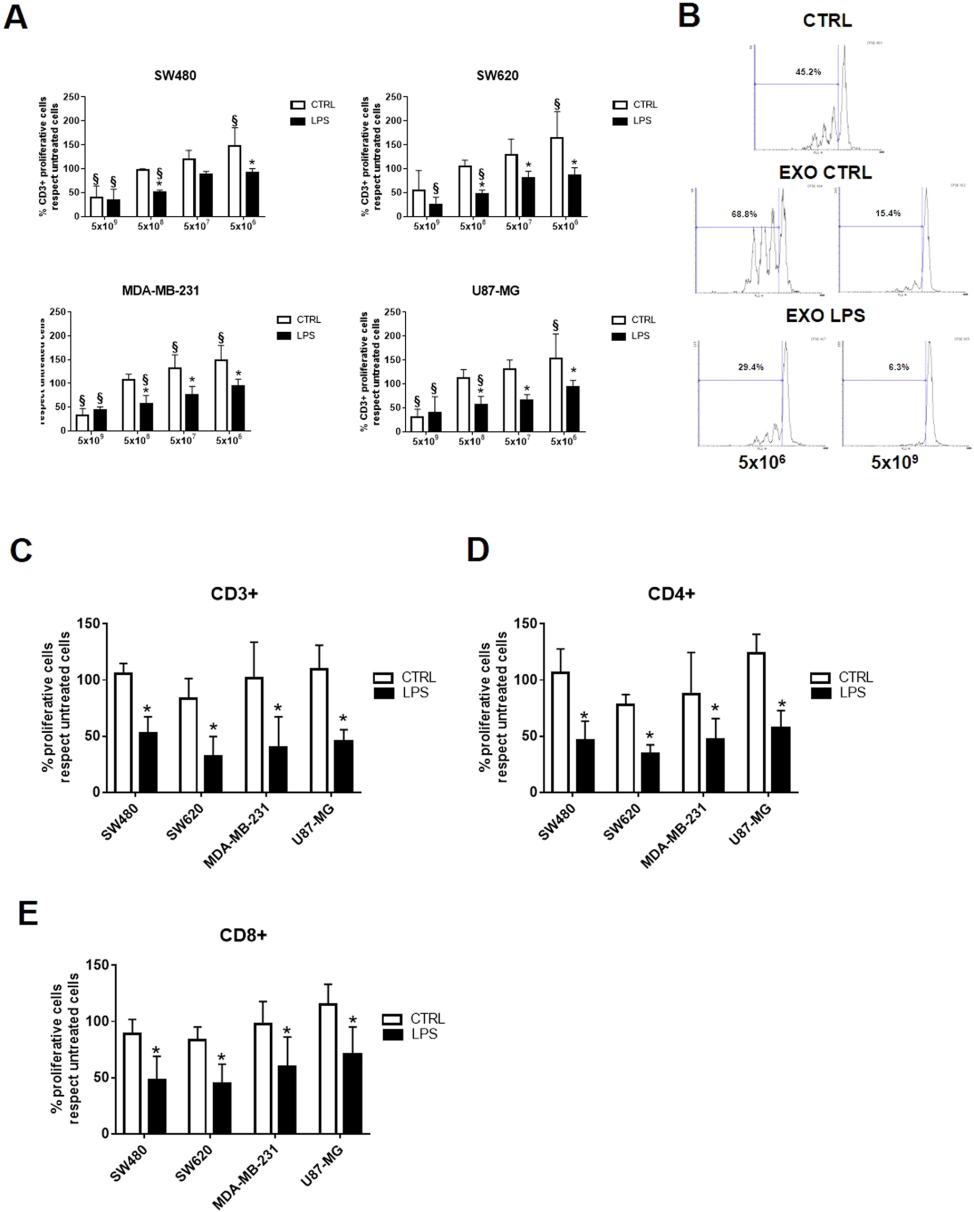
Exosomes released after LPS treatment inhibit T cell proliferation in a dose-dependent manner. (**A**) CFSE-labeled PBMCs isolated from healthy donors were pre-treated for 24 hours with scalar doses (from 5 × 10^6^ to 5 × 10^9^) of exosomes released by unstimulated (CTRL, white column) or LPS-activated tumor cells (LPS, black column). Histograms show the percentage of CD3+ T cells in proliferation, assigning the value of 100% to the proliferation activity of untreated cells. Data are shown as mean (n = 5) ± SD. ^§^*P* < 0.05 compared to untreated CD3+ T cells; *P < 0.05 compared to the proliferation of CD3+ T cells treated with exosomes released by unstimulated tumor cells; (**B**) Representative cytometry CFSE histograms show the proliferative fraction of untreated (CTRL) and exosomes-treated (EXO CTRL and EXO LPS) CD3+ T cells. CFSE-labeled PBMCs were pretreated for 24 hours with 5 × 10^8^ exosomes and histograms show, within the PBMCs, the fraction of proliferating CD3+ (**C**), CD4+ (**D**) and CD8+ (**E**) T cells, considering the proliferation of untreated cells as 100%. Data are shown as mean (n = 6) ± SD. *P < 0.05.

To determinate whether activation of TLR4 expressed on tumor cells influences the immune-modulatory properties of released exosomes, CFSE-labelled PBMCs, isolated from healthy donors, were incubated for 24 hours with scalar doses (from 5 × 10^6^ to 5 × 10^9^) of exosomes and then stimulated with anti-CD3/-CD28. We found that only exosomes released upon LPS treatment, up to 5 × 10^8^, significantly inhibited CD3+ T cell proliferation in a dose-dependent manner. In all other cases, only the highest dose (5 × 10^9^) of exosomes released by the untreated cells was able to inhibit the proliferation of CD3+ T cells. With lower doses, the inhibitory effect of the exosomes released by the unstimulated cells disappeared, while a stimulation effect on T cell proliferation appeared (Fig. 3A,B).

In order to evaluate the effect of the exosomes on the proliferation of different T cell subtypes, the PBMCs were incubated with 5 × 10^8^ exosomes and labeled for the expression of CD4 and CD8. As the dose of exosomes to use in the experiments, the smallest dose able to induce a different effect on the proliferation of T lymphocytes between control and LPS-treated exosomes was chosen, based on previous results.

We found that only exosomes released after LPS treatment were able to significantly inhibit the proliferation of both, CD4+ (Fig. 3D) and CD8+ (Fig. 3E) T cells.

### Internalization of exosomes by immune cells

**Figure 4:**
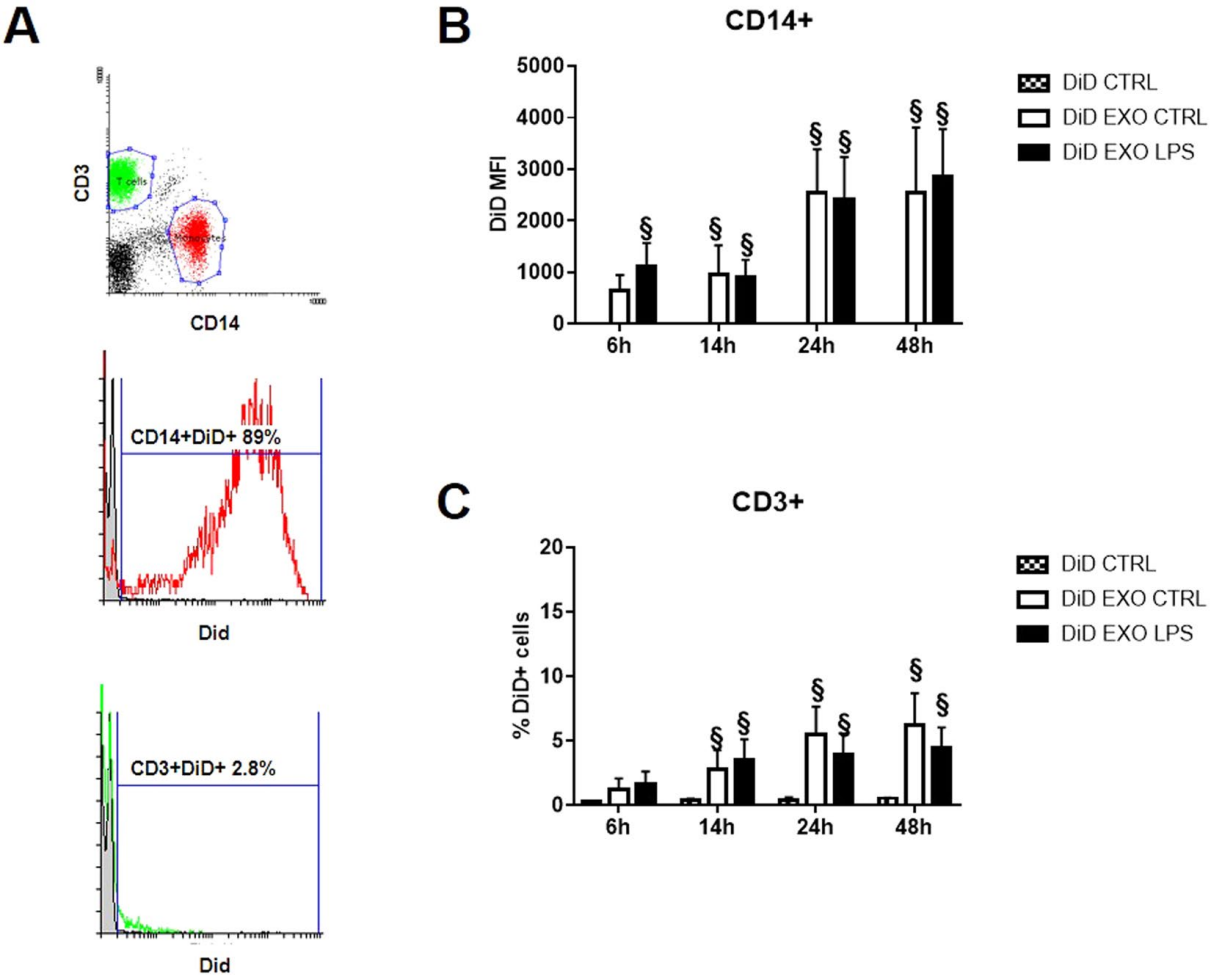
Tumor-derived exosomes were internalized by monocytes, rather than by T cells. (**A**) DiD-labelled exosomes were incubated with PBMCs and their uptake by CD14+ monocytes or CD3+ T cells was measured at different time points by flow cytometric analysis. In representative cytometry histograms, PBMCs cells were gated based on CD14 (red) or CD3 expression (green). Histograms show the DiD mean fluorescence intensity (MFI) of CD14+ monocytes (**B**) and the percentage of DiD+ CD3+ cells. Data are shown as mean (n = 4) ± SD. ^§^*P* < 0.05.

To investigate cellular interactions responsible for the inhibitor effect of exosomes on T cell proliferation, we stained the vesicles with DiD dye and monitored their uptake by T cells and monocytes isolated from PBMCs. As reported in Fig. 4A, CD14+ monocytes quickly and efficiently internalized DiD-labelled exosomes. After 6 hours, about 90% of monocytes were positive for DiD (data not shown) and their mean fluorescence intensity (MFI) increased in a time-depend manner (Fig. 4B). Surprisingly, CD3+ T cells slightly internalized tumor exosomes, even after 48 hours of co-incubation (Fig. 4C). No differences were observed between exosomes released by the different cell lines (data not shown). Exosomes released by cells after LPS stimulation were internalized with the same kinetics observed for control exosomes. Our results suggested that the internalization of tumor-derived exosomes was not necessary for deliver the signals that result in inhibition of T cells proliferation. They also suggest that surface contact might be sufficient for inducing the changes observed.

### Exosomes released upon LPS treatment modulate the production of inflammatory proteins by monocytes

In order to evaluate the effect of the tumor-derived exosomes on monocytes, purified CD14+ cells were incubated with 5 × 10^8^ exosomes and the production of inflammatory proteins (sE-Selectin, GM-CSF, IFNα, IFNγ, IL-1α, IL-1β, IL-4, IL-6, IL-8, IL-10, IL-12p70, IL-13, IL-17A/CTLA-8, IP-10/CXCL10, MCP-1/CCL2, MIP-1α/CCL3, MIP-1β/CCL4, sP-Selectin, TNFα) were analyzed on cell supernatants by multiplex ELISA. The levels of sE-Selectin, GM-CSF, IFNα, IFNγ, IL-4, IL-12p70, IL-13 and IL-17A were below the level of detection in all the samples analyzed (data not shown).

**Figure 5:**
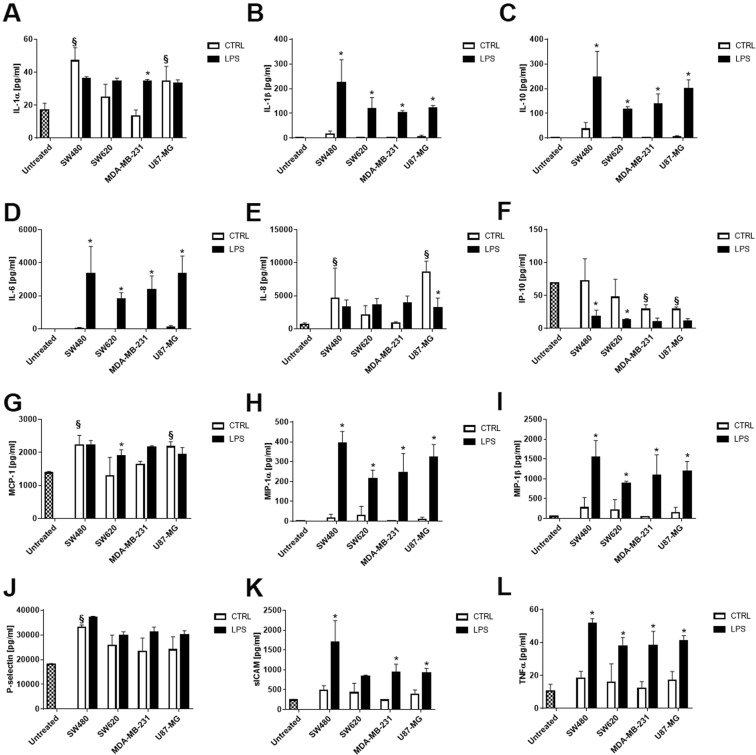
Tumor-derived exosomes modulate the production of inflammatory proteins by monocytes. Monocytes were incubated for 48 hours with exosomes released by unstimulated (CTRL, white column) or LPS-activated tumor cells (LPS, black column) and the production of inflammatory proteins was quantified in supernatants by ELISA Bio-plex assay system. Data are shown as mean (n = 3) ± SD. ^§^*P* < 0.05 compared to untreated monocytes; *P < 0.05 compared to monocytes treated with exosomes released by unstimulated tumor cells.

SW480- and U87-MG-derived exosomes induce the release of IL-1α (Fig. 5A), IL-8 (Fig. 5E) and MCP-1 (Fig. 5G) by monocytes, while exosomes released from all tumor cell lines after LPS treatment trigger the production of IL1β (Fig. 5B), IL-10 (Fig. 5C), IL-6 (Fig. 5D), MIP-1α (Fig. 5H), MIP-1β (Fig. 5I) and TNF-α (Fig. 5L) cytokines. The release of slCAM was increased in monocytes treated with LPS-stimulated SW480-, SW620- and U87-MG- derived exosomes (Fig. 5K). On the contrary, MDA-MB-231 and U87-MG- derived exosomes and SW480- and SW620- derived exosomes released after LPS treatment inhibited the production of IP-10 (Fig. 5F).

Finally, to ensure that the inflammatory proteins measured were not contaminants derived from the isolation process, their presence was explored directly in purified exosomes. The results indicate that all analytes were below the level of detection or expressed at low levels respect the concentration measured on monocytes supernatants (data not shown).

### Expression of TGFβ on exosomes surface and its effect on regulatory T cells expansion

**Figure 6:**
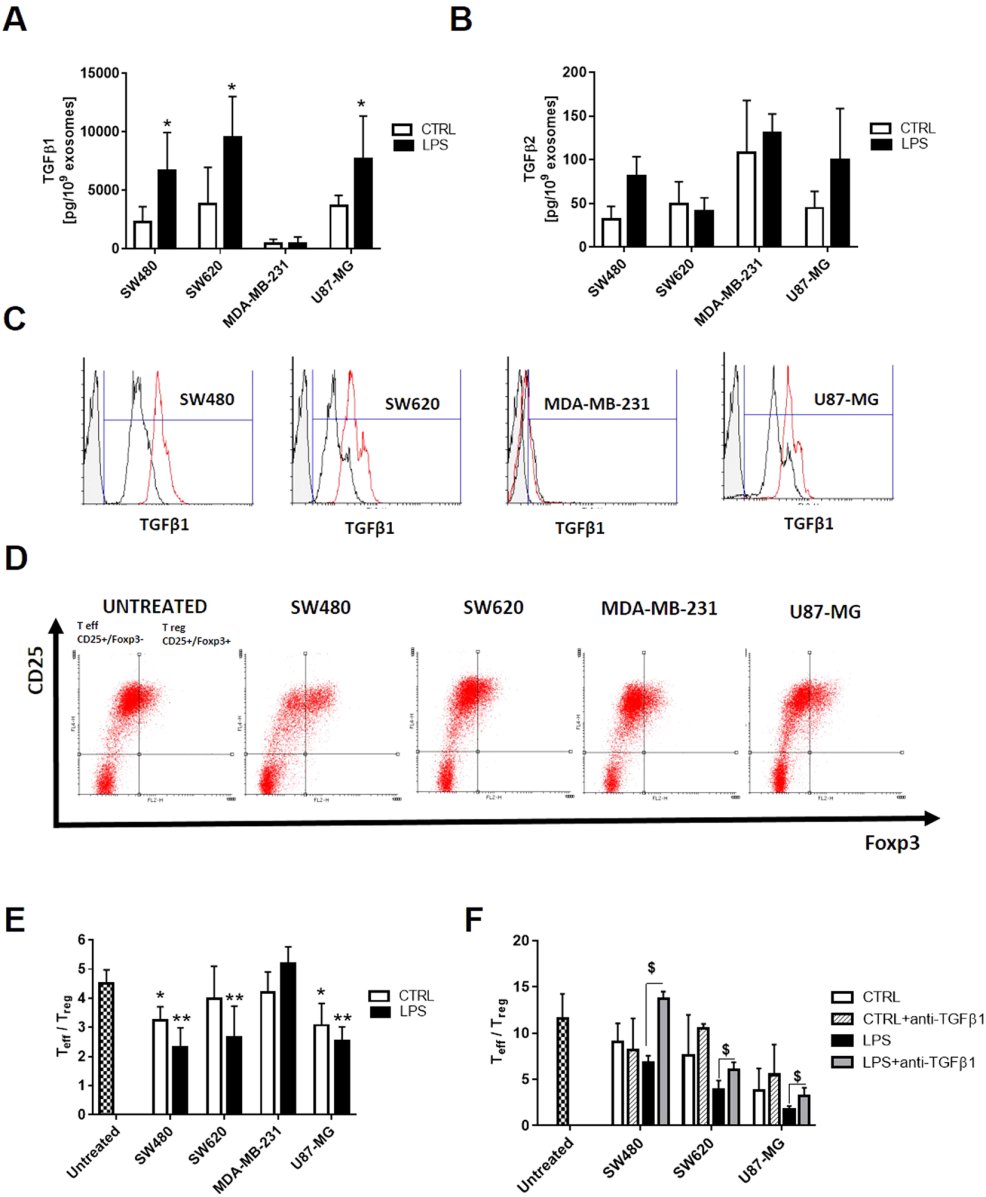
Expression of TGFβ on the surface of exosomes and its effect on regulatory T cells expansion. The expression of TGFβ1 (**A**) and TGFβ2 (**B**) on the surface of exosomes were evaluated using the TGF-β Magnetic Luminex Performance Assay on exosomes released by unstimulated (CTRL, white column) o LPS-activated tumor cells (LPS, black column). Data are shown as mean (n = 8) ± SD. *Difference with exosomes released by unstimulated tumor cells, *P* < 0.05. (**B**) Exosomes were coupled to ExoFlow beads, stained with monoclonal antibodies against TGFβ1 and analyzed by FACS. Representative plots of the FACS analyses for staining of control (black line) and of LPS-derived exosomes (red line) are shown. (**D–F**) CD4+ T cells, isolated from PBMCs by negative selection, were stimulated with anti-CD3, anti-CD28 and IL-2 in the presence of exosomes released by unstimulated (CTRL, white column) o LPS-activated tumor cells (LPS, black column). The percentage of effector (CD4+/CD25−/FoxP3−) or regulatory T cells (CD4+/CD25+/FoxP3+) was determined by fluorescence activated flow cytometry (FACS) on day 4. Representative plots for CD25 and Foxp3 staining are shown and histograms represent the ratio between the percentage of effector and regulatory T cells (T_eff_/T_reg_). In a separate set of experiments, exosomes were pre-treated with neutralizing anti-TGFβ1 antibody. Data are shown as mean (n = 6) ± SD. *difference with the untreated cells, *P* < *0.05*; **difference with untreated cells, *P* < *0.005*, ^$^difference with exosomes treated cells released by LPS-activated cells.

To investigate the molecular mechanisms underlying the inhibitory effects of exosomes released by tumor cells upon TLR4 activation by LPS, we examined whether TGFβ was present on exosome surface, as this cytokine is strongly implicated in mechanisms of immune evasion and may be responsible for the antiproliferative effect observed in our study. We evaluated the expression of the three different isoforms of TGFβ by multiplex assay, finding that only TGFβ1 and TGFβ2 were measurable in our exosomal samples, while TGFβ3 was undetectable (data not shown). Specifically, TGFβ1 was expressed at a very low level on the surface of exosomes released by MDA-MB-231 as compared to the other cells lines (Fig. 6A). Interestingly, we found that the expression of TGFβ1 was increased in exosomes released by SW480, SW620 and U87-MG cells upon TLR4 activation by LPS, while no differences was observed for MDA-MB-231-derived exosomes. The expression of TGFβ2 on exosome surface was not influenced by cell TLR4 activation by LPS (Fig. 6B).

The expression of TGFβ1 on the exosomes surface was also confirmed by FACS analysis of exosomes coupled to Exo-Flow beads (Fig. 6C).

In addition, we hypothesized that TGFβ1 expressed on the surface of exosomes could also induce the conversion of CD4+ CD25− T cells into Foxp3-expressing regulatory T cells. Stimulation by exosomes secreted by untreated SW480 and U87-MG increased the percentage of CD4+/CD25+/Foxp3+ cells in the PBMC population (Fig. 6D,E) and this stimulatory effect becomes statistically more significant with exosomes released by cells upon TLR4 activation. Interestingly, only exosomes produced by SW620 cells upon TLR4 activation become capable of inducing conversion to regulatory T cells. Moreover, the pre-incubation of exosomes released by SW480, SW620 and U87-MG LPS-activated cells with neutralizing anti-TGFβ1 antibody resulted in a decrease of the percentage of CD4+/CD25+/Foxp3+ cells, suggesting that *in vitro* induction of regulatory T cells by exosomes is mediated by TGFβ1 (Fig. 6F).

### Expression of NKG2D ligands on the surface of exosomes and modulation of NKG2D expression on NK and CD8+ T cells

**Figure 7:**
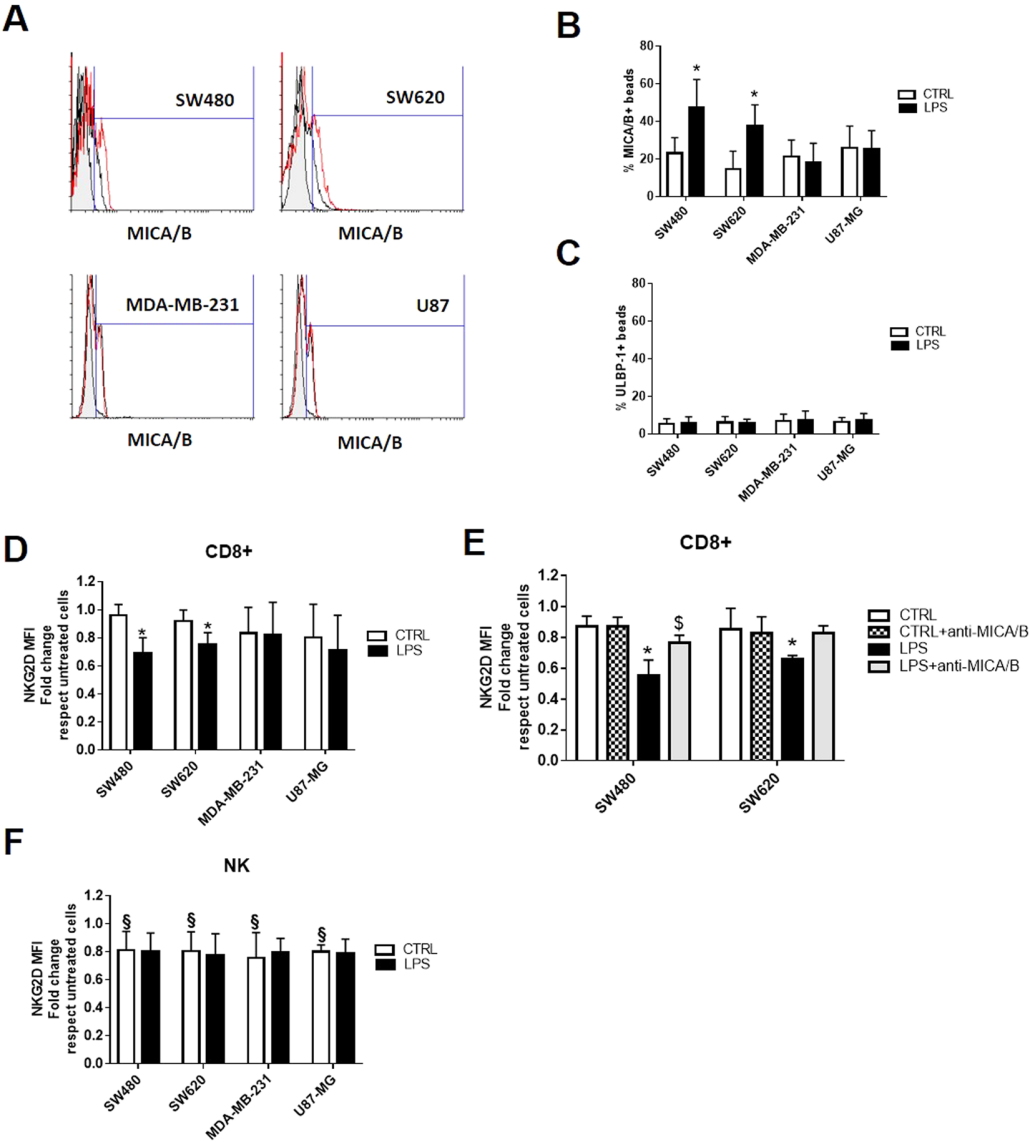
Expression of NKG2D ligands on the surface of exosomes and modulation of NKG2D expression on NK and CD8+ T cells. Exosomes were coupled to ExoFlow beads, stained with monoclonal antibodies either against MICA/B or ULBP-1 and analyzed by FACS. (**A**) Representative plots of the FACS analyses for MICA/B staining of control (black line) and of LPS-derived exosomes (red line) are shown. The histograms represent the percentages of MICA/B- (**B**) and ULBP-1 (**C**) -positive beads bound to the exosomes released by unstimulated (CTRL, white column) o LPS-activated cells (LPS, black column). Data are shown as mean (n = 6); bars, SD; *difference with exosomes released by unstimulated tumor cells, *P* < 0.05. PBMCs or isolated CD16+ CD56+ NK cells were treated for 24 hours with exosomes released by unstimulated (CTRL, white column) o LPS-activated tumor cells (LPS, black column). In a separate set of experiments, exosomes were pre-treated with neutralizing anti-MICA/B antibody (**E**). Expression of NKG2D by CD8+ (**D,E**) and NK cells (**F**) was analyzed by FACS. Data are shown as mean (n = 6); bars, SD; ^§^difference with untreated cells; *difference with cells treated with exosomes released by unstimulated tumor cells, *P* < 0.05, ^$^difference with exosomes treated cells released by LPS-activated cells, *P* < 0.05.

There is mounting evidence showing that tumor cells escape immune surveillance by the release of soluble NKG2D ligands, which trigger a general downregulation of the NKG2D receptor on NK cells and CD8+ T cells^21^. The expression of MICA/B and ULBP-1, a NKG2D ligands, on the surface of exosomes coupled to Exo-Flow beads was evaluated by FACS analysis. As shown in the representative plot of the FACS analysis, tumor exosomes expressed, even if at low levels, MICA/B (Fig. 7A,B), but not ULPB-1 (Fig. 7C). Interestingly, the expression of MICA/B was increased following activation of TLR4 with LPS only in the exosomes released by SW480 and SW620. Moreover, they were able to reduce the expression of NKG2D in CD8+ T lymphocytes (Fig. 7D). The pre-incubation with neutralizing anti-MICA antibody of exosomes released by LPS-activated SW480 and SW620 cells resulted in an increased expression of NKG2D in CD8+ cells, although this effect was statistically significant only for SW480-derived exosomes (Fig. 7E). In contrast, the expression of NKG2D in purified NK cells was slightly reduced by exosomes released from all tumor cell lines, but no further effect was observed after activation of TLR4 with LPS. (Fig. 7F).

### Expression of miR-21, miR-34a and miR-155 in tumor-derived exosomes

**Figure 8:**
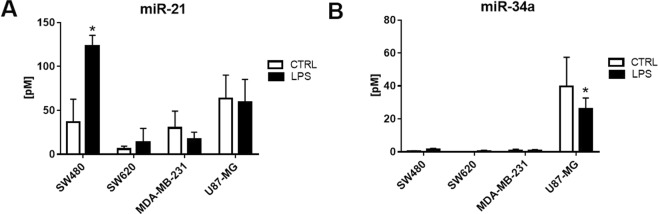
Expression of miR-21 and miR-34a in tumor-derived exosomes. The concentration of miR-21 (**A**) and miR-34a (**B**) was measured in exosomes produced by unstimulated (CTRL, white column) o LPS-activated tumor cells (LPS, black column) by qRT–PCR. Data are presented as mean (n = 3); bars, SD, *difference with exosomes released by unstimulated tumor cells, *P* < 0.05.

Several specific miRNAs have recently emerged as important regulators of immune-cell function in the context of different solid tumors^22^. Among these, miR-21, miR-155, and miR-34a are mediators of immune suppression, as they promote the expression of immunosuppressive factors. To analyze the expression of these miRNAs, total RNA from tumor-derived exosomes released by cells upon TLR4 activation was extracted. The expression of miRNA-21 (Fig. 8A) was significantly higher in exosomes produced by SW480 pre-treated with LPS compared to those of untreated cells, while no difference was observed for exosomes produced by other cell lines. On the other hand, we found miR-34a only in exosomes produced by U87-MG and its expression was significantly decreased after treatment with LPS (Fig. 8B). Finally, exosomes produced by all the cell lines analyzed were did not show expression of the miRNA-155 (data not shown).

## Discussion

It has been well established that the immune system plays an important role in monitoring tumor development and progression. However, a number of mechanisms have been discovered that allow tumors to escape from the host immune defenses by a process known as “immune evasion”. Among various molecular and/or genetic changes that tumors induce in the immune cells, the production of exosomes is emerging as novel mechanism of immune suppression^23^. Specifically, it has been described that tumor-derived-exosomes (TEX) impair dendritic cell differentiation and maturation, decrease the cell cytotoxicity of NK cells, push the differentiation of myeloid precursor cells toward MDSCs, reduce T cells response and induce regulatory T and B cells^3,8^.

Until now, many studies have been directed to investigate the mechanisms by which tumor-derived exosomes are able to support tumor growth but little is known about the effect that the microenvironment or the activation of cancer cells exert on the composition and functionality of TEXs released in the environment. It has been proposed that the content and number of exosomes produced may change if the cells are exposed to different stressors or stimuli, and that exosomes produced by the same cells can contain different constituents. A recent study has shown that exosomes mediate hypoxia-dependent intercellular signaling of glioblastoma multiforme, a highly malignant brain tumor, since the profile of the proteome and mRNA species found in the vesicles closely reflect the oxygenation status of glioma cells in patients’ tumors^24^.

To further investigate those functions, we evaluated the effect of toll-like receptor 4 (TLR4) activation on the immune modulatory properties of exosomes released by tumor cells. TLR4, belongs to the family of pattern recognition receptors (PRRs), highly conserved receptors that recognize pathogen-associated molecular patterns (PAMPs) and damage-associated molecular pattern (DAMPs), thus representing the first line of defense against infections^25^. Accumulating evidence demonstrated that TLRs may promote carcinogenesis through pro-inflammatory, anti-apoptotic, proliferative and profibrogenic signals in either the tumor microenvironment or tumor cells themselves^26^. These effects can be either induced directly in TLR-expressing target cells, or mediated by TLR-induced cytokines^26^. Specifically, tumor-promoting functions of TLR4 are best established in the colon^27^, liver^28^, breast^14^, pancreas^29^ and skin^30^ cancers. Likewise, it has been reported that TLR4 is expressed on SW480^31^, SW620^32^, MDA-MB-231^33^ and U87-MG^34^ tumor cells and its activation increases migration^33–36^ and tumorigenesis^14^ of the cell lines used in this study.

Our data suggest that the activation of TLR4 on tumor cells did not influence the number or size of released TEXs, instead, it increased their immunosuppressive potential. As already reported in the literature^37,38^, we found that exosomes secreted by untreated tumor cells are able to inhibit T cells proliferation, however the effect occurs only with high-dose treatments, while low doses stimulate the proliferation of T cells. Instead, exosomes produced by tumor cells after TLR4 activation were always immunosuppressive, suggesting that the treatment influences TEX cargo. The molecular profile show that TEX may express a dual capability of mediating either immune suppression or immune stimulation, presumably depending upon the microenvironment^3^. The immunostimulatory properties of TEXs are probably due to the presence of tumor-associated antigens (TAA) and costimulatory molecules on TEX surface^9^, which stimulate the immune response. It has been reported that exosomes produced by lymphoma cells after a heat-shock contained high levels of Hsp70 and were reported to stimulate a direct Th1-polarized immune response in a MHC-independent manner in autologous and allogeneic murine models^39^.

Upon secretion, exosomes are able to fuse with the plasma membranes of recipient cells to deliver their content into the cytoplasm. Alternatively, proteins present on their surface can bind cell surface receptors on target cells and influence intracellular signaling^40^.

We reported that tumor-derived exosomes were efficiently internalized by CD14+ monocytes and were able to modulate their production of inflammatory proteins. Specifically, exosomes produced by tumor cells after TLR4 activation strongly induce the release of pro-inflammatory cytokines and chemokines in monocytes. It is widely recognized that abnormalities in cytokine expression are implicated in tumorigenesis as they promote growth, reduce apoptosis and facilitate invasion and metastasis of cancer cells^41,42^. Moreover, it has been reported that tumor‐derived EVs induce in primary monocytes an activated phenotype, which is also observed in tumor‐associated macrophages^43^.

We observed that while monocytes rapidly take up exosomes, T cells did not internalize TEXs, even after 48 hours of co-incubation. According with other studies in which the Authors show that T lymphocytes, unlike other mononuclear cells, do not internalize exosomes^44^, our data suggest that TEXs deliver signals to receptors present on the T cell surface. It has been previously proposed that exosomes produced by cancer cells may induce regulatory T cells growth through the TGF-β1 expressed on their surface^45,46^. Our data demonstrate that TLR4 activation increases the expression of TGFβ1 on TEX surface and promotes the expansion of regulatory T cells, which in turn may be responsible for the reported antiproliferative effects of TEXs on T cells. We also found that exosomes secreted by the breast cancer cell line MDA-MB-231, expressed TGF-β1 at a very low level, as compared to exosomes released by the other cells lines, even after TLR4 cell activation, and fail to induce regulatory T cells.

The production of NKG2D-ligand-bearing exosomes is a newly described mechanism for cancer cell immune evasion^21,47,48^. We found that exosomes released by all cell lines analyzed express on their surface MICA/B, a NKG2D ligand, although only in colorectal cancer cells-derived exosomes TLR4 activation increased its expression. It is therefore possible to hypothesize that colorectal cancer cells are more sensitive to the activation of TLR4. In fact, a correlation between the chronic activation of TLR4 and the progress of CRC through the release of immunosuppressive factors which promote tumor escape has been widely shown^24,37,38^.

Increasing evidences suggest that exosomal microRNAs transported by cancer cells can be affect other cells in the local microenvironment leading to reprogramming of the target cell transcriptome and influencing cancer progression in a paracrine manner^49^. MiR-21 and miR-155 are well-characterized oncomiRs that promote both, cancer development and metastasis by targeting numerous mRNAs. In contrast, miR-34a has been shown to suppress cancer growth and metastasis by inducing apoptosis, cell cycle arrest and senescence. We found that the expression of miR-21 was increased in SW480-derived exosomes after TLR4 activation. It has been proposed that in the early stage of inflammation, pri-miR-21 exerts pro-inflammatory effects and polarizes macrophages towards the pro inflammatory M1 phenotype^50^. Moreover, miR-21 present in cancer cell-secreted exosomes can be transferred to surrounding immune cells and can bind to endosomal Toll-like receptors. By binding to TLRs, it induces cytokines secretion by the immune cells, leading to a prometastatic inflammatory response that ultimately may lead to cancer growth and metastasis^51^.

In conclusion, the present study demonstrates that the activation of TLR4 contributes to cancer progression by promoting the release of more effective immunosuppressive exosomes, which allow tumor cells to escape immune surveillance. As a matter of fact, exosomes released by tumor cells after LPS treatment can inhibit the proliferation of T lymphocytes more effectively. Further investigations will be needed to identify the common mechanisms underlying the observed effects.

## Supplementary information

Supplementary Figure 1

## References

[1] Hessvik NP, Llorente A (2018). Current knowledge on exosome biogenesis and release. Cell. Mol. Life Sci..

[2] Samanta S (2017). Exosomes: new molecular targets of diseases. Acta Pharmacol. Sin..

[3] Whiteside, T. L. Tumor-Derived Exosomes and Their Role in Cancer Progression. In *Advances in clinical chemistry* **74**, 103–141 (2016). 10.1016/bs.acc.2015.12.005 PMC5382933 27117662

[4] Lobb RJ, Lima LG, Möller A (2017). Exosomes: Key mediators of metastasis and pre-metastatic niche formation. Semin. Cell Dev. Biol..

[5] Greening DW, Gopal SK, Xu R, Simpson RJ, Chen W (2015). Exosomes and their roles in immune regulation and cancer. Semin. Cell Dev. Biol..

[6] Czernek L, Düchler M (2017). Functions of Cancer-Derived Extracellular Vesicles in Immunosuppression. Arch. Immunol. Ther. Exp. (Warsz)..

[7] Dörsam, B., Reiners, K. S. & von Strandmann, E. P. Cancer-derived extracellular vesicles: friend and foe of tumour immunosurveillance. *Philos. Trans. R. Soc. Lond. B. Biol. Sci*. **373** (2018). 10.1098/rstb.2016.0481 PMC5717436 29158311

[8] Whiteside TL (2016). Exosomes and tumor-mediated immune suppression. J. Clin. Invest..

[9] Whiteside TL (2017). Exosomes in Cancer: Another Mechanism of Tumor-Induced Immune Suppression. Advances in experimental medicine and biology.

[10] Shalapour S, Karin M (2015). Immunity, inflammation, and cancer: an eternal fight between good and evil. J. Clin. Invest..

[11] Thuringer D (2011). Transactivation of the epidermal growth factor receptor by heat shock protein 90 via Toll-like receptor 4 contributes to the migration of glioblastoma cells. J. Biol. Chem..

[12] Gong W (2013). Invasion potential of H22 hepatocarcinoma cells is increased by HMGB1-induced tumor NF-κB signaling via initiation of HSP70. Oncol. Rep..

[13] Wang C (2012). HMGB1 was a pivotal synergistic effecor for CpG oligonucleotide to enhance the progression of human lung cancer cells. Cancer Biol. Ther..

[14] Yang H (2014). Toll-like receptor 4 prompts human breast cancer cells invasiveness via lipopolysaccharide stimulation and is overexpressed in patients with lymph node metastasis. PLoS One.

[15] Oblak A, Jerala R (2011). Toll-like receptor 4 activation in cancer progression and therapy. Clin. Dev. Immunol..

[16] Ran S (2015). The Role of TLR4 in Chemotherapy-Driven Metastasis. Cancer Res..

[17] Tang X, Zhu Y (2012). TLR4 signaling promotes immune escape of human colon cancer cells by inducing immunosuppressive cytokines and apoptosis resistance. Oncol. Res..

[18] Korneev KV (2017). TLR-signaling and proinflammatory cytokines as drivers of tumorigenesis. Cytokine.

[19] Domenis R (2018). Pro inflammatory stimuli enhance the immunosuppressive functions of adipose mesenchymal stem cells-derived exosomes. Sci. Rep..

[20] Domenis Rossana, Cesselli Daniela, Toffoletto Barbara, Bourkoula Evgenia, Caponnetto Federica, Manini Ivana, Beltrami Antonio Paolo, Ius Tamara, Skrap Miran, Di Loreto Carla, Gri Giorgia (2017). Systemic T Cells Immunosuppression of Glioma Stem Cell-Derived Exosomes Is Mediated by Monocytic Myeloid-Derived Suppressor Cells. PLOS ONE.

[21] Mincheva-Nilsson L, Baranov V (2014). Cancer exosomes and NKG2D receptor–ligand interactions: Impairing NKG2D-mediated cytotoxicity and anti-tumour immune surveillance. Semin. Cancer Biol..

[22] Paladini L (2016). Targeting microRNAs as key modulators of tumor immune response. J. Exp. Clin. Cancer Res..

[23] Whiteside Theresa (2016). Tumor-Derived Exosomes and Their Role in Tumor-Induced Immune Suppression. Vaccines.

[24] Kucharzewska P (2013). Exosomes reflect the hypoxic status of glioma cells and mediate hypoxia-dependent activation of vascular cells during tumor development. Proc. Natl. Acad. Sci. USA.

[25] Molteni M, Gemma S, Rossetti C (2016). The Role of Toll-Like Receptor 4 in Infectious and Noninfectious Inflammation. Mediators Inflamm..

[26] Pradere J-P, Dapito DH, Schwabe RF (2014). The Yin and Yang of Toll-like receptors in cancer. Oncogene.

[27] Yesudhas D, Gosu V, Anwar MA, Choi S (2014). Multiple Roles of Toll-Like Receptor 4 in Colorectal Cancer. Front. Immunol..

[28] Yang J, Li M, Zheng QC (2015). Emerging role of Toll-like receptor 4 in hepatocellular carcinoma. J. Hepatocell. carcinoma.

[29] Vaz J, Andersson R (2014). Intervention on toll-like receptors in pancreatic cancer. World J. Gastroenterol..

[30] Burns EM, Yusuf N (2014). Toll-like receptors and skin cancer. Front. Immunol..

[31] Tang X-Y, Wang H, Zhu Y-Q, Wei B (2010). Expression and Functional Research of TLR4 in Human Colon Carcinoma. Am. J. Med. Sci..

[32] Huang H-Y (2014). The TLR4/NF-κB signaling pathway mediates the growth of colon cancer. Eur. Rev. Med. Pharmacol. Sci..

[33] Li J (2017). TLR4 Promotes Breast Cancer Metastasis via Akt/GSK3β/β-Catenin Pathway upon LPS Stimulation. Anat. Rec..

[34] Sarrazy V (2011). TLR4 signal transduction pathways neutralize the effect of Fas signals on glioblastoma cell proliferation and migration. Cancer Lett..

[35] Zhou B (2011). Activation of PAR2 or/and TLR4 promotes SW620 cell proliferation and migration via phosphorylation of ERK1/2. Oncol. Rep..

[36] Zhu G (2016). LPS Upregulated VEGFR-3 Expression Promote Migration and Invasion in Colorectal Cancer via a Mechanism of Increased NF-κB Binding to the Promoter of VEGFR-3. Cell. Physiol. Biochem..

[37] Ye S-B (2014). Tumor-derived exosomes promote tumor progression and T-cell dysfunction through the regulation of enriched exosomal microRNAs in human nasopharyngeal carcinoma. Oncotarget.

[38] Domenis R (2017). Systemic T Cells Immunosuppression of Glioma Stem Cell-Derived Exosomes Is Mediated by Monocytic Myeloid-Derived Suppressor Cells. PLoS One.

[39] Chen W (2006). Efficient induction of antitumor T cell immunity by exosomes derived from heat-shocked lymphoma cells. Eur. J. Immunol..

[40] Kalluri R (2016). The biology and function of exosomes in cancer. J. Clin. Invest..

[41] Burkholder B (2014). Tumor-induced perturbations of cytokines and immune cell networks. Biochim. Biophys. Acta - Rev. Cancer.

[42] Landskron G, De la Fuente M, Thuwajit P, Thuwajit C, Hermoso MA (2014). Chronic inflammation and cytokines in the tumor microenvironment. J. Immunol. Res..

[43] Gärtner K (2018). Tumor-derived extracellular vesicles activate primary monocytes. Cancer Med..

[44] Muller L (2017). Human tumor-derived exosomes (TEX) regulate Treg functions via cell surface signaling rather than uptake mechanisms. Oncoimmunology.

[45] Clayton A, Mitchell JP, Court J, Mason MD, Tabi Z (2007). Human tumor-derived exosomes selectively impair lymphocyte responses to interleukin-2. Cancer Res..

[46] Yen E-Y, Miaw S-C, Yu J-S, Lai I-R (2017). Exosomal TGF-β1 is correlated with lymphatic metastasis of gastric cancers. Am. J. Cancer Res..

[47] Linnane MD (2018). Down-Modulate NKG2D Expression Human Tumor-Derived Exosomes Human Tumor-Derived Exosomes Down-Modulate NKG2D Expression. J Immunol Ref. J. Immunol..

[48] Lundholm M (2014). Prostate Tumor-Derived Exosomes Down-Regulate NKG2D Expression on Natural Killer Cells and CD8+ T. Cells: Mechanism of Immune Evasion..

[49] Bell E, Taylor MA (2017). Functional Roles for Exosomal MicroRNAs in the Tumour Microenvironment. Comput. Struct. Biotechnol. J..

[50] Sheedy FJ (2015). Turning 21: Induction of miR-21 as a Key Switch in the Inflammatory Response. Front. Immunol..

[51] Fabbri M (2012). MicroRNAs bind to Toll-like receptors to induce prometastatic inflammatory response. Proc. Natl. Acad. Sci..

