# Supplementary material for: Toll-like Receptor-4 Activation Boosts the Immunosuppressive Properties of Tumor Cells-derived Exosomes
Source: Sci Rep. 2019 Jun 11;9:8457. doi: 10.1038/s41598-019-44949-y (PMC6560033; doi:10.1038/s41598-019-44949-y)
Supplement: Supplementary file 1 — Supplementary Figure 1 [file 41598_2019_44949_MOESM1_ESM.pdf]

Toll-like Receptor-4 Activation Boosts the Immunosuppressive Properties of Tumor Cells-derived Exosomes

Rossana Domenis, Adriana Cifù, Daniele Marinò, Martina Fabris, Kayvan R. Niazi, Patrick Soon-Shiong, Francesco Curcio

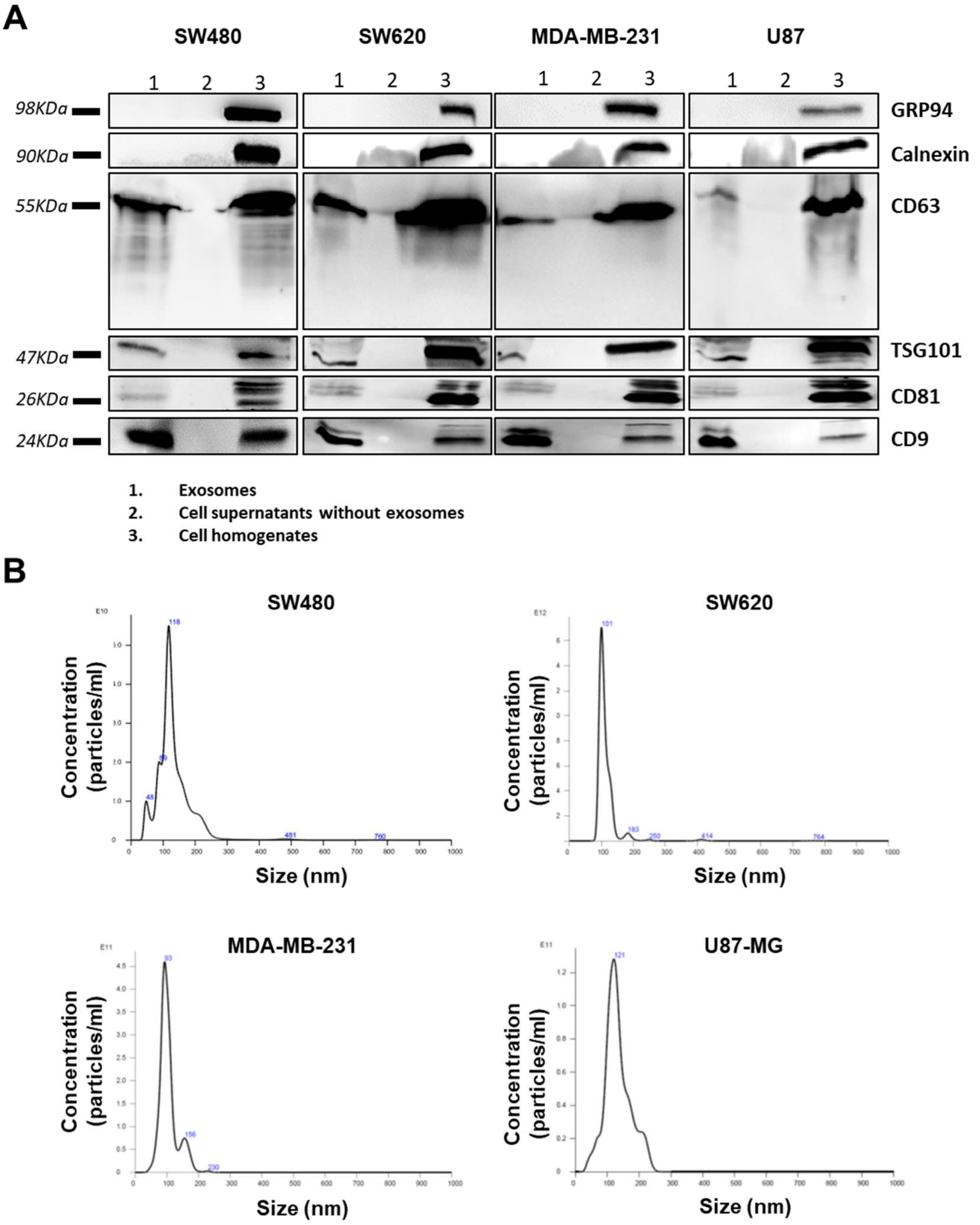

Supplementary Figure 1. Immunoblotting and particles size analysis of tumor-cells derived exosomes

(A) Immunoblotting analysis of exosomes, Exoquick-derived supernatants and cells homogenates probed for the indicated proteins. (B) Representative NTA graphs of frequency size distribution of particles.
